# Extension of T_2_ Hyperintense Areas in Patients With a Glioma: A Comparison Between High‐Quality 7 T MRI and Clinical Scans

**DOI:** 10.1002/nbm.5316

**Published:** 2025-01-28

**Authors:** Bárbara Schmitz‐Abecassis, Ivo Cornelissen, Robin Jacobs, Jasmin A. Kuhn‐Keller, Linda Dirven, Martin Taphoorn, Matthias J. P. van Osch, Johan A. F. Koekkoek, Jeroen de Bresser, the 7T MRI Neuro‐Oncology consortium, Monique Baas‐Thijssen, Christa Benit, Jeroen de Bresser, Marike Broekman, Linda Dirven, Daniëlle van Dorth, Lara Fritz, Melissa Kerkhof, Johan A. F. Koekkoek, Rishi Nandoe‐Tewarie, Matthias J. P. van Osch, Bárbara Schmitz‐Abecassis, Martin J. B. Taphoorn, Maaike Vos

**Affiliations:** ^1^ Department of Radiology Leiden University Medical Center Leiden The Netherlands; ^2^ Medical Delta South‐Holland The Netherlands; ^3^ Department of Neurology Leiden University Medical Center Leiden The Netherlands; ^4^ Department of Neurology Haaglanden Medical Center The Hague The Netherlands; ^5^ Department of Neurology Alrijne Hospital Leiden The Netherlands

**Keywords:** glioma, T_2 _hyperintense area, tumor extension, tumor visualization

## Abstract

Gliomas are highly heterogeneous and often include a nonenhancing component that is hyperintense on T_2_ weighted MRI. This can often not be distinguished from secondary gliosis and surrounding edema. We hypothesized that the extent of these T_2_ hyperintense areas can more accurately be determined on high‐quality 7 T MRI scans. We investigated the extension, volume, and complexity (shape) of T_2_ hyperintense areas in patients with glioma on high‐quality 7 T MRI scans compared to clinical MRI scans. T_2_ hyperintense areas of 28 patients were visually compared and manually segmented on 7 T MRI and corresponding clinical (1.5 T/3 T) MRI scans, and the volume and shape markers were calculated and subsequently compared between scans. We showed extension of the T_2_ hyperintense areas via the corpus callosum to the opposite hemisphere in four patients on the 7 T scans that was not visible on the clinical scan. Furthermore, we found a significantly larger volume of the T_2_ hyperintense areas on the 7 T scans compared with the clinical scans (7 T scans: 28 mL [12.5–59.1]; clinical scans: 11.9 mL [11.8–56.6]; *p* = 0.01). We also found a higher complexity of the T_2_ hyperintense areas on the 7 T scans compared with the clinical scans (convexity, solidity, concavity index and fractal dimension [*p* < 0.001]). Our study suggests that high‐quality 7 T MRI scans may show more detail on the exact extension, size, and complexity of the T_2_ hyperintense areas in patients with a glioma. This information could aid in more accurate planning of treatment, such as surgery and radiotherapy.

## Graphical abstract

Exact glioma extension and shape might be underestimated on clinical MRI scans. High‐quality 7 T MRI suggests more detail on extension, size, and complexity of T_2_ hyperintense areas. Having more detailed information on tumor extension, size, and complexity of T_2_ hyperintense areas in patients with a glioma could aid in more accurate planning of treatment, such as surgery and radiotherapy.

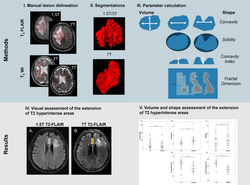

**Abbreviations**
CNR: contrast‐to‐noise ratio
Gy: gray
IDH: isocytrate dehydrogenase
IQR: interquartile range
MRI: magnetic resonance imaging
PCV: procarbazine + CCNU (lomustine) + vincristine
SNR: signal‐to‐noise ratio
(T_2_WI): T_2_ weighted imaging

## 1 Introduction

Gliomas are the most common primary malignant brain tumor and are diffuse and heterogeneous in their morphology [1]. They are known to grow along white matter tracts and usually include nonenhancing components as well as possible secondary gliosis (related to treatment effects and secondary ischemia) and the usual surrounding edema [2, 3, 4]. Patients with glioma often undergo MRI scans for the purpose of diagnosis, treatment planning, and follow‐up [5]. Standard clinical practice includes the acquisition of T_2_ weighted imaging (T_2_WI) and T_2_ fluid attenuated inversion recovery (T_2_‐FLAIR) [6]. The T_2_WI scans allow visualization of fluid rich compartments as well as abnormal tissue, as these usually have longer T_2_ relaxation times and thus will appear hyperintense. T_2_‐FLAIR allows portraying abnormal tissue in a similar way, but with the difference that any free moving fluid is suppressed. Combined, these clinical images allow portrayal of important details on tumor morphology. However, distinguishing between nonenhancing tumor components and secondary changes (such as edema and gliosis), which all appear as T_2_ hyperintense areas can be challenging [6]. Information on the exact extent of these could aid in more accurate planning of tumor‐directed treatment, such as surgery and radiotherapy.

Ultra‐high field 7‐T MRI benefits from an increase in contrast and signal to noise ratio (CNR and SNR, respectively), allowing the acquisition of higher resolution images with more contrast between subcomponents of tumors [7]. The higher resolution potential is harnessed by yielding images with smaller voxel size, resulting in less partial volume effect and thus more tissue specific representation per voxel. For patients with glioma in clinical practice this could mean obtaining images with enhanced tumor visualization regarding tissue boarders and extension in a reasonable clinical acquisition time [8]. The more limited resolution of current clinical MRI scans might underestimate the tumor extension along white matter tracts, which could influence the planning of radiotherapy and surgical resection.

The value of using ultra‐high field 7 T MRI for patients with glioblastoma has been studied before; for example, Regnery et al. have demonstrated that 7 T T_2_‐FLAIR images may enhance the delineation of organs at risk for radiotherapy planning, such as the hippocampus, which could help to preserve long‐term cognitive function [9]. Moreover, they found an increase of signal‐to‐noise ratio (SNR), but a decrease in contrast‐to‐noise ratio (CNR). A smaller gross tumor volume of the T_2_ hyperintense area was also found in the 7 T T_2_‐FLAIR images. Although their sequence duration was longer than common in current clinical practice, it still proved to be clinically feasible. This study showed preliminary evidence of the benefit of higher quality images for this patient population, specifically concerning radiotherapy planning. However, several important other features, such as tumor shape, were not considered. Shape would be a particularly interesting feature to explore, given the suggestion that gliomas may grow along white matter tracts, potentially shaping the complexity of these lesions [3, 4]. Moreover, the previous study only looked at patients with glioblastoma, whereas visualization of T_2_ hyperintense areas is at least as clinically relevant in generally noncontrast enhancing lower grade gliomas. Given that ultra‐high field 7 T MRI has the potential to acquire higher quality images at reasonable scanning times, we wanted to investigate if these benefits can enhance glioma tumor visualization in general and help in estimating its extension and complexity. We therefore aimed to investigate the extension, volume and shape of T_2_ hyperintense areas in patients with a glioma on high‐quality 7 T MRI scans compared with clinical MRI scans.

## 2 Methods

### 2.1 Patient Inclusion

Patients from the Leiden University Medical Center and Haaglanden Medical Center were prospectively included for this study between March 2021 and May 2023 when there was a high suspicion of having a glioma or a confirmed histopathological diagnosis. Other inclusion criteria included a Karnofsky Performance Status score ≥ 70 and no MRI contraindications. The study was approved by the local ethics committee and all patients gave written informed consent prior to participation.

### 2.2 Data Acquisition

Clinical MRI scans, obtained at either 1.5 T or 3 T, were collected as part of patients' standard clinical care, adhering to routine clinical guidelines; 3 T T_2_WI multislice were acquired on a Philips Ingenia 3 T MRI scanner (Philips Healthcare, Best, The Netherlands) with TR = 4490 ms TE = 80 ms, voxel size = 0.4 × 0.5 × 3 mm and FOV = 220 × 175 × 50. Total acquisition time was 01:57 min.1.5 T T_2_‐FLAIR multislice were acquired on a Siemens MAGNETOM Avanto 1.5‐T scanner (Siemens, Erlangen, Germany) with TR = 7500 ms, TE = 105 ms, voxel size = 0.4 × 0.4 × 5 mm and FOV = 230 × 230 × 144 mm. Total acquisition time was 02:08 min.

MRI scans were acquired using a Philips Achieva 7 T MRI scanner (Philips Healthcare, Best, The Netherlands) with a dual‐transmit and a 32‐channel receive head coil (Nova Medical Inc, Wilmington, MA, USA). The 3D T_2_WI was acquired with TR = 3000 ms, TE = 278 ms, voxel size = 0.75 × 0.75 × 0.75 mm, FOV = 250 × 250 × 190 mm, and the total acquisition time was 04:06 min. The 3D T_2_‐FLAIR was acquired with TR = 8000 ms, TE = 256 ms, voxel size = 0.7 × 0.7 × 0.7 mm, FOV = 240 × 209 × 190 mm, and the total acquisition time was 05:12 min.

High‐quality 7 T MRI and clinical MRI scans (1.5 T or 3 T) were acquired, on average, within (mean) 5 days (±5.3 standard deviation) of each other.

### 2.3 Visual Assessment of the T_2_ Hyperintense Areas

Firstly, a systematic visual assessment of the T_2_ hyperintense areas was performed by evaluating side by side the high‐quality and clinical MRI scans for all 28 patients. The goal was to compare the range of extension of the T_2_ hyperintense areas with a special focus on the involvement of neighboring anatomical structures. Such structures also served as anatomical landmarks for a fair comparison between the clinical and 7 T images acquired. Examples of considered structures include the corpus callosum, the basal ganglia, brain stem, and white matter tracts. In cases where the tumor was located in the center of the brain (close to the brain stem), and artifacts in the high‐quality MRI scans impaired proper visualization, the clinical scans were used to determine the border of the T_2_ hyperintense area. The visual assessment was performed by a radiologist in training (IC) under supervision of an experienced neuro‐radiologist (JB).

### 2.4 Segmentation of the T_2_ Hyperintense Areas

Segmentations of the T_2_ hyperintense areas were performed by two investigators on T_2_WI, and when not available, on the T_2_‐FLAIR. To minimize learning effects between delineations performed on the clinical images and those on the 7 T images, all delineations were first completed on the clinical images, followed by the 7 T images. This process was accomplished under supervision of IC and JB. Although the segmentations were done by two observers, the methodology employed and the radiological supervision was the same. This means that a methodological consensus was reached between the radiologist (in training) and the two investigators before performing the segmentations and that the segmentations were visually checked in a consensus meeting with the experienced neuro‐radiologist. Areas were defined as T_2_ hyperintense when there was a clear and solid hyperintense area including the tumor core, peritumoral edema, and/or nonenhancing tumor components. Other T_2_ hyperintense brain changes such as resection cavities, infarctions, and age‐related white matter hyperintensities were excluded from the segmentations. Finally, in cases where the border between the T_2_ hyperintense lesion and eventual white matter hyperintensities could not be clearly distinguished, we defined what would most probably be the T_2_ hyperintense area and its respective border. The same strategy was used for both clinical and 7 T scans to result in similar segmentation results.

### 2.5 Volume and Shape of the T_2_ Hyperintense Areas

Volume and shape markers were computed for the T_2_ hyperintense areas. Shape markers, including solidity, convexity, concavity index, and fractal dimension, were calculated based on the convex hull, volume, and surface areas of the segmented lesions, similarly to a method previously used for white matter hyperintensities [9]. In cases where patients had T_2_ hyperintense areas appearing continuous on the 7 T high‐quality scans, but fragmented in the clinical scans (i.e., more than one area), the largest area was chosen for the shape analysis.

### 2.6 Statistical Analysis

To evaluate differences in volume and shape of the T_2_ hyperintense areas between the high‐quality 7 T MRI scans compared with the clinical MRI scans, we conducted a paired sample Wilcoxon Signed‐rank test, given the nonnormal distribution of the data.

As a sensitivity analysis, we repeated the same analysis after excluding areas smaller than 10 cm^3^. This threshold was selected to minimize potential segmentation errors, which tend to be more pronounced in smaller areas because of the lower number of voxels included and does especially influence shape markers.

The significance level was set at *p* ≤ 0.05. Statistical analysis was performed using IBM SPSS version 29 (Chicago, IL).

## 3 Results

### 3.1 Patient Inclusion

In total, we recruited 28 patients with a glioma (Table 1) of whom the majority had a glioblastoma, isocytrate dehydrogenase (IDH) wild‐type (71%), whereas the remaining patients had an astrocytoma, IDH‐mutant (18%), or (suspected) oligodendroglioma, IDH‐mutant, 1p/19q codeleted (11%). Most patients had undergone some form of tumor targeted treatment, with only 18% being included before first surgery. At the time of the MRI scans, five patients were using dexamethasone.

**TABLE 1:** Clinical characteristics of the glioma patients (*n* = 28).

**Patient demographics**	
Age, mean ± standard deviation	58 ± 12
Female	13 (46%)
Male	15 (53%)
**Diagnosis**	
Glioblastoma, IDH‐wildtype	20 (71%)
Astroctyoma, IDH‐mutant, grade 4	1 (4%)
Astrocytoma, IDH‐mutant, grade 3	1 (4%)
Astrocytoma, IDH‐mutant, grade 2	3 (10%)
Oligodendroglioma, IDH‐mutant, 1p/19q codeleted	2 (7%)
Suspected oligodendroglioma	1 (4%)
**Tumor location**	
Temporal	11 (40%)
Frontal	13 (46%)
Parietal	2 (7%)
Other	2 (7%)
**Intervention**	
No intervention	5 (18%)
Surgery	23 (82%)
Partial resection	16 (57%)
Total resection	1 (4%)
Biopsy	6 (2%)
Radiotherapy	15 (54%)
Photon therapy	14 (50%)
Proton therapy	1 (4%)
Total dose 30 Gy	2 (7%)
40 Gy	2 (7%)
60 Gy	11 (39%)
**Adjuvant chemotherapy**	**19 (68%)**
Temozolomide	17 (61%)
Temozolomide and lomustine	1 (4%)
PCV	1 (4%)
**Total daily use of dexamethasone**	**5 (18%)**
1 mg	3 (11%)
4 mg	1 (4%)
6 mg	1 (4%)

*Note:* Variables represent number of patients (*n*) and the percentage of the total patient population (%).

Abbreviations: IDH, isocitrate dehydrogenase; PCV, procarbazine, CCNU (lomustine), vincristine.

### 3.2 Visual Assessment of the Extension of T_2_ Hyperintense Areas

**FIGURE 1:**
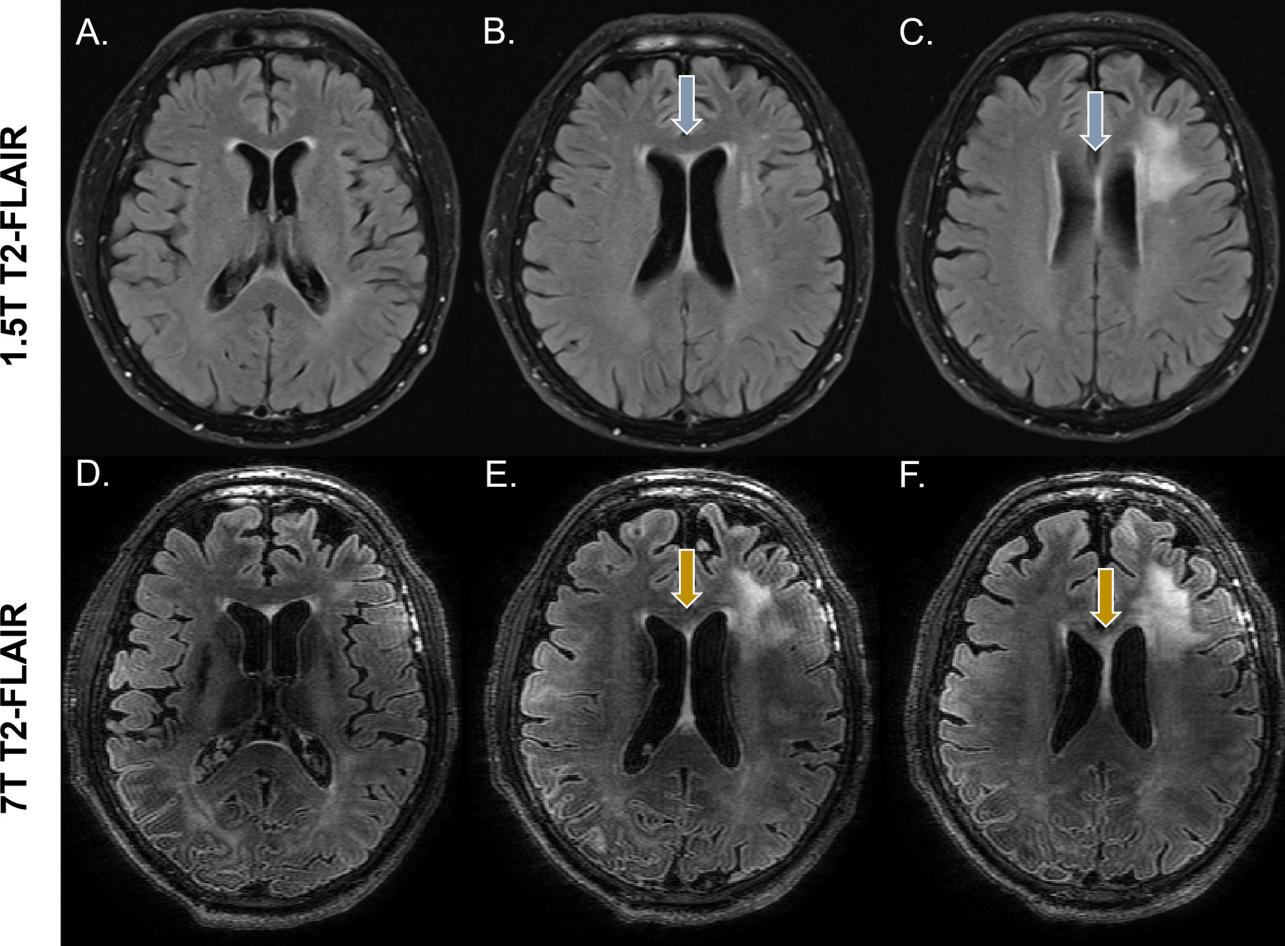
Visual assessment of tumor extension represented on T_2_‐FLAIR images in a patient with glioblastoma, IDH‐wild‐type, showing three representative slices of the tumor in A–C for the clinical 1.5 T T_2_‐FLAIR and in D‐F for the high‐quality 7 T T_2_‐FLAIR. In this case, the corpus callosum involvement is better observed in the high‐quality 7 T images. Especially, the connectivity between the T_2_ hyperintense area in the corpus callosum and the lesions can be more clearly followed on the high‐quality 7 T image compared with the clinical scan.

**FIGURE 2:**
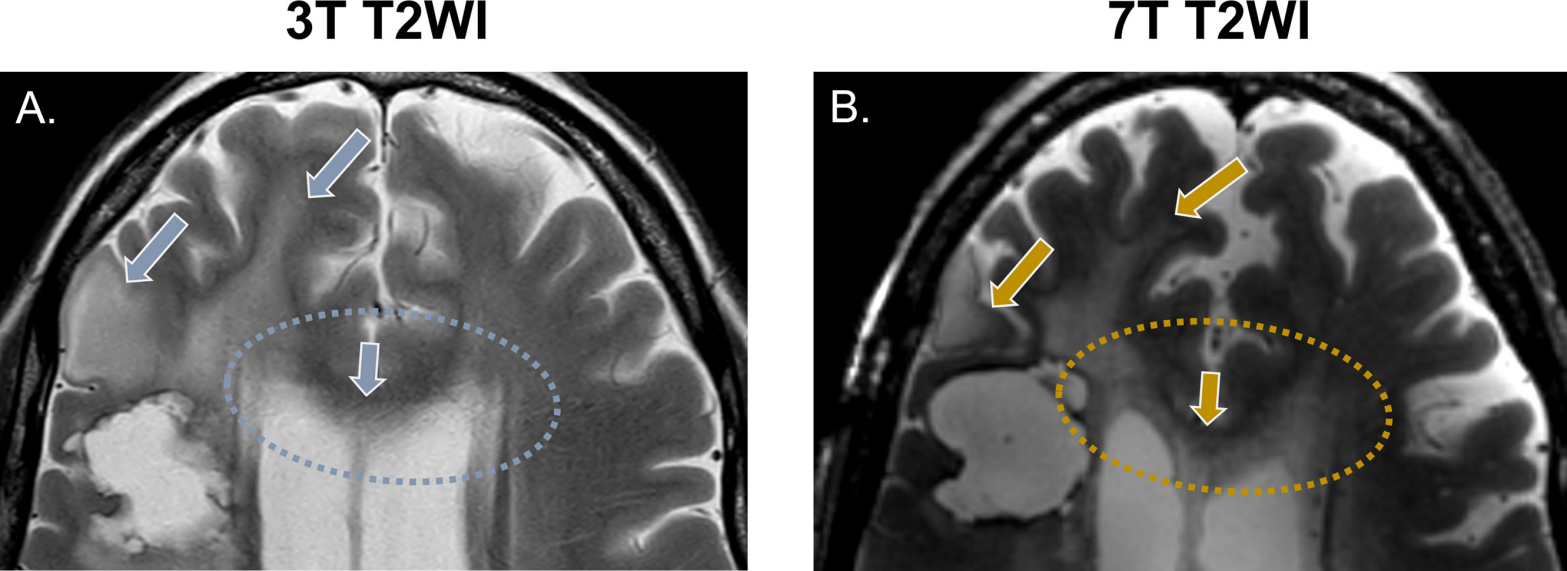
Visual assessment of tumor extension on T_2_‐weighted images. The presented case is from a patient after partial resection of a glioblastoma, IDH‐wild‐type, indicating T_2_ hyperintensities in the corpus callosum. While in A., the clinical scan cannot clearly illustrate its structure due to partial volume with the ventricles, in B., the high‐resolution scan shows a clear involvement of the corpus callosum, as well as how it connects to the T_2_ hyperintense areas in both hemispheres.

**FIGURE 3:**
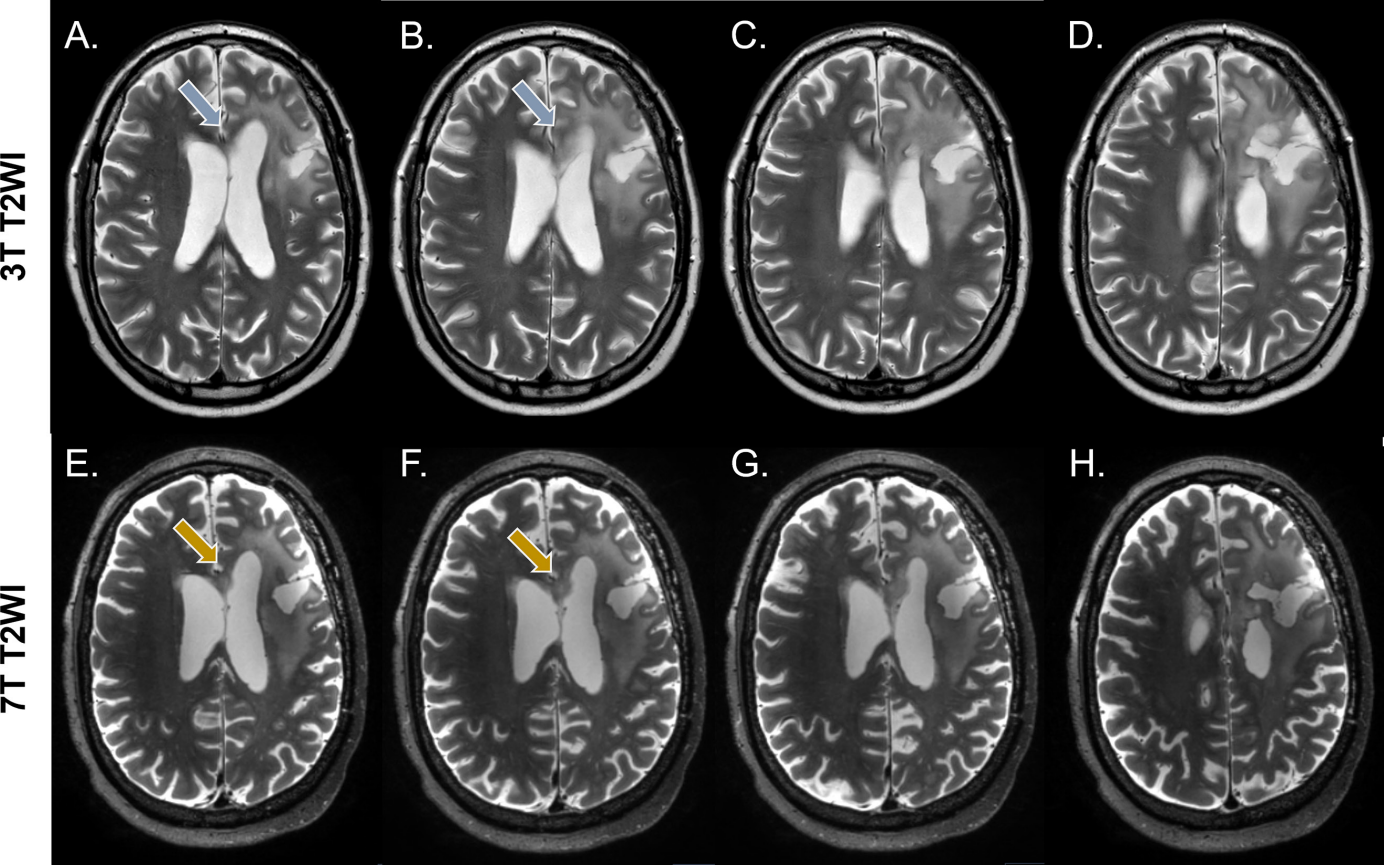
Visual assessment of tumor extension using T_2_‐weighted images. The example presented is from a patient after partial resection of a diffuse astrocytoma, IDH‐mutant WHO grade 2. In A. and B., it is challenging to determine whether the T_2_ hyperintensities indicated by the blue arrow are due to the partial volume effects from the ventricles or whether they suggest the involvement of the corpus callosum, despite the T_2_ hyperintense tumor area appearing to connect with that region in C. and D. The high‐resolution 7 T images in the bottom row, E. and H., provide a clear view of the T_2_ hyperintense areas in this region, indicated by the yellow arrows, where the hyperintense area can be distinctly seen as a shape of its own. In G., a distinct connection of this area with the primary tumor area on the right hemisphere can be seen. In H., the tumor extension to the right hemisphere is evident from the periventricular T_2_ hyperintense area, which can be clearly distinguished from the ventricle. On the other hand, in D., this can also be clearly observed on the clinical scan.

**FIGURE 4:**
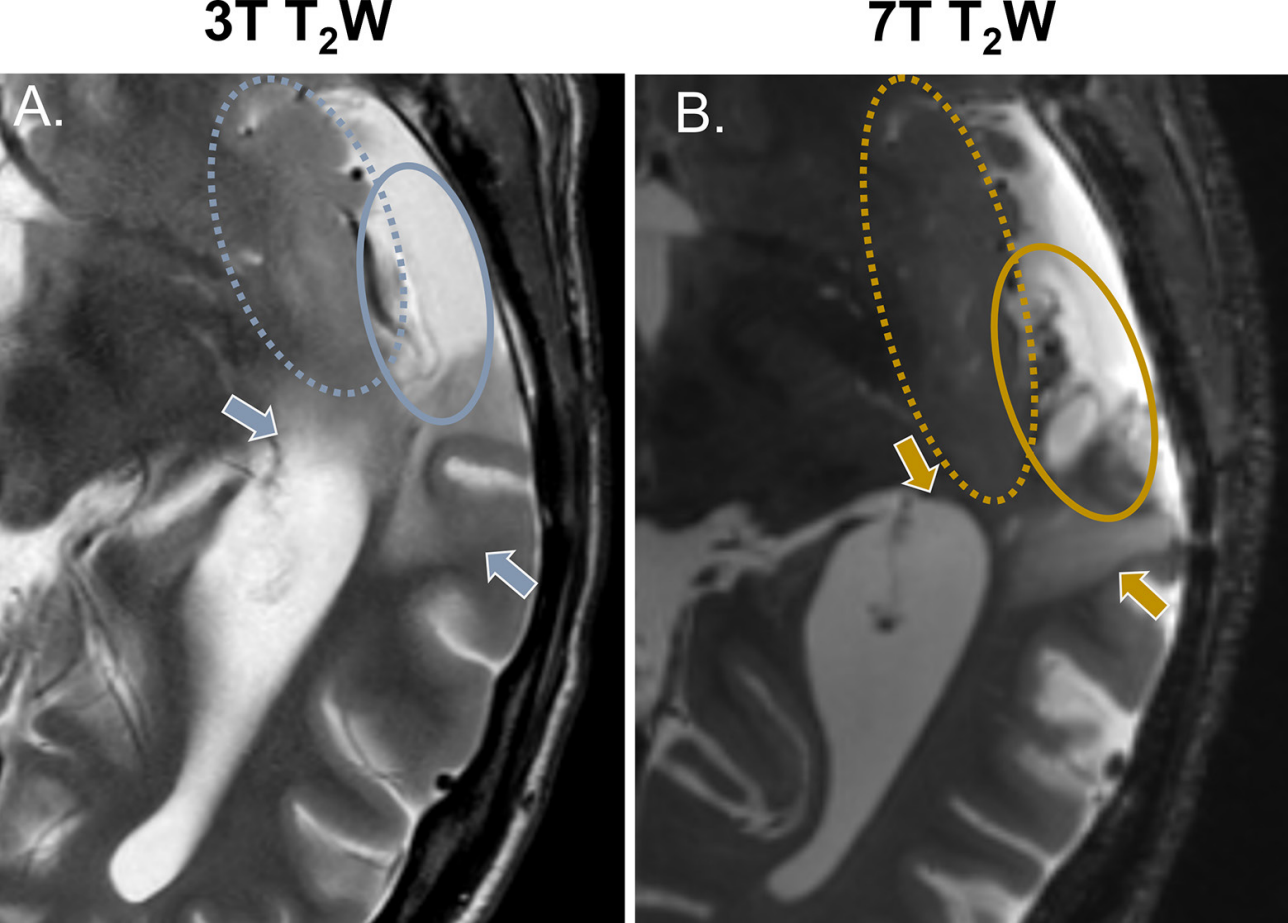
Visual assessment of tumor characteristics and boundaries using T_2_‐weighted images. An example patient after partial resection of a diffuse astrocytoma, IDH mutant WHO grade 2 is presented. In A., the clinical image illustrates, with blue arrows, how the boundaries between tissues are blurred in this case. In contrast, in B., the high‐quality 7 T image clearly depicts these boundaries, indicated by the yellow arrows. Differences in how characteristics within the resection cavity can be visualized are also noticeable, indicated by the full‐line ellipses in blue and yellow. The dotted ellipses in both images indicate the involvement of the insular cortex, which can actually be more clearly seen on the clinical scan. This is a result from signal drop in the deep regions of the brain that can occur with 7 T imaging.

**FIGURE 5:**
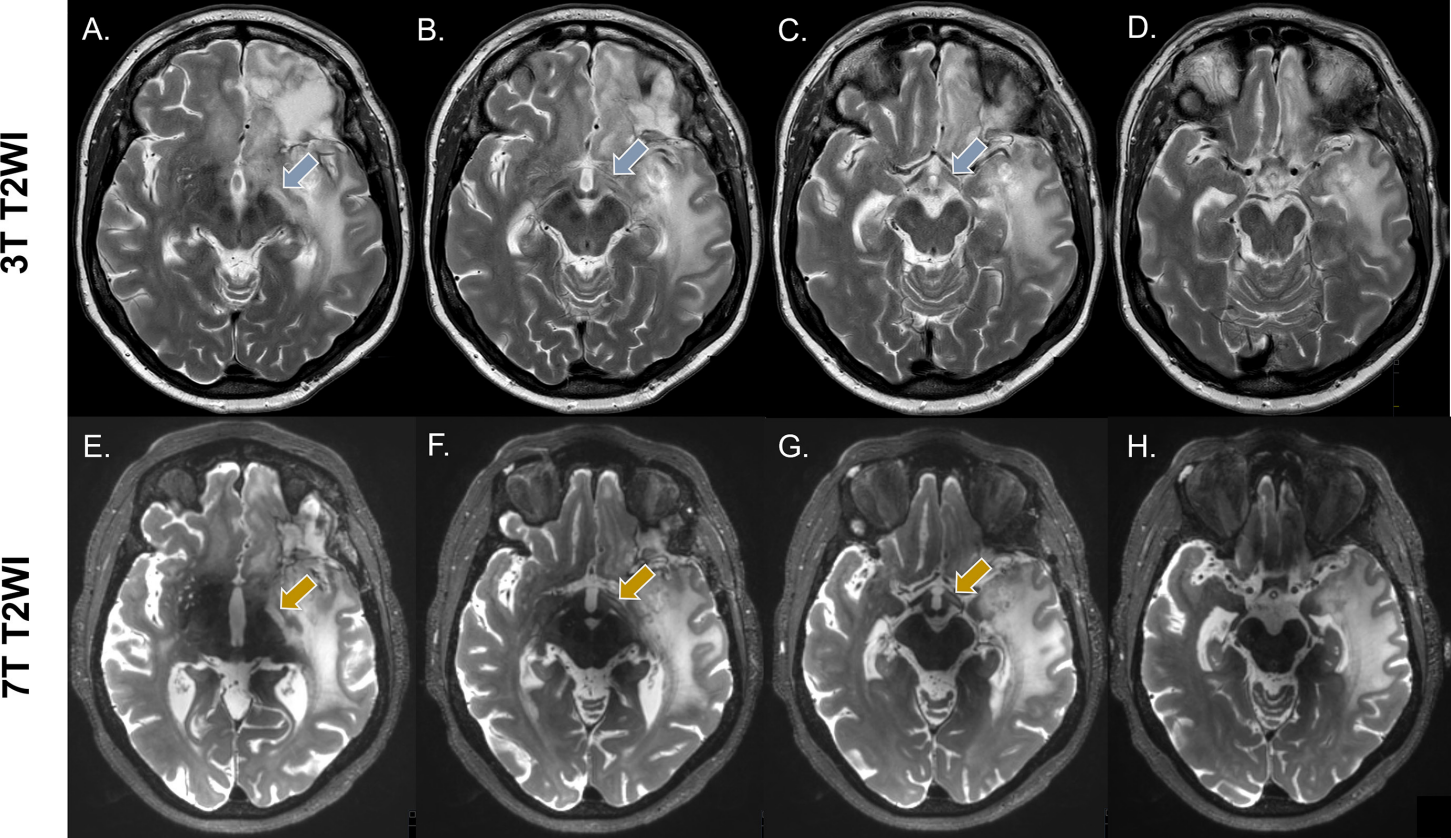
Visual assessment of tumor extension to the left optic tract using T_2_‐weighted images. An example of a patient with a glioblastoma, IDH‐wild‐type in whom there is a suspicion of involvement of the left optic tract in the tumor pathology. Whereas a T_2_ hyperintense area can be seen on the clinical scans, the connection between the left optic tract and the lesion can be better followed on the high‐resolution 7 T MRI scans.

The comparison between the high‐quality and clinical MRI scans yielded a few visual differences that were observed when systematically going through all cases. Namely, it was observed that in 10 patients there was involvement of the corpus callosum. In four of these patients, this involvement could be more clearly discerned on the high‐quality 7 T images. In the remaining six, the corpus callosum involvement was equally well observed on both high‐quality and clinical MRI scans. In the cases where the high‐quality 7 T images provided a more clear involvement, we could trace the connection of the corpus callosum hyperintensity to the primary hyperintense area (Figures 1, 2, 3). High‐quality 7 T scans also allowed for enhanced tumor visualization, such as tissue boundaries, for example, between ventricles and white matter and between tumor lesions and healthy appearing tissue (Figure 4). However, we also noticed that in the deep areas of the brain, the clinical 3 T scans outperformed the 7 T scans. This can be observed in Figure 4, where the involvement of the insular cortex is shown to be more hyperintense on the clinical image (Figure 4A). The involvement of fiber tracts in the T_2_ hyperintense area, could be better visualized on the high‐quality 7 T images. An example can be seen in Figure 5, where involvement of the optical tract can be seen, just as that we could better exclude the involvement of other smaller and finer structures such as the optical chiasm. Another interesting example in Figure S1 shows a patient with a glioblastoma where most likely Wallerian degeneration is present secondary to the tumor pathology, which was virtually invisible on the clinical scan, whereas its presence can be clearly visualized on the high‐quality image. Overall, these examples show that high‐quality 7 T MRI scans in some cases and certain areas of the brain provide improved visualization of T_2_ hyperintense areas compared with standard clinical scans.

### 3.3 Volume and Shape Assessment of the Extension of T_2_ Hyperintense Areas

**FIGURE 6:**
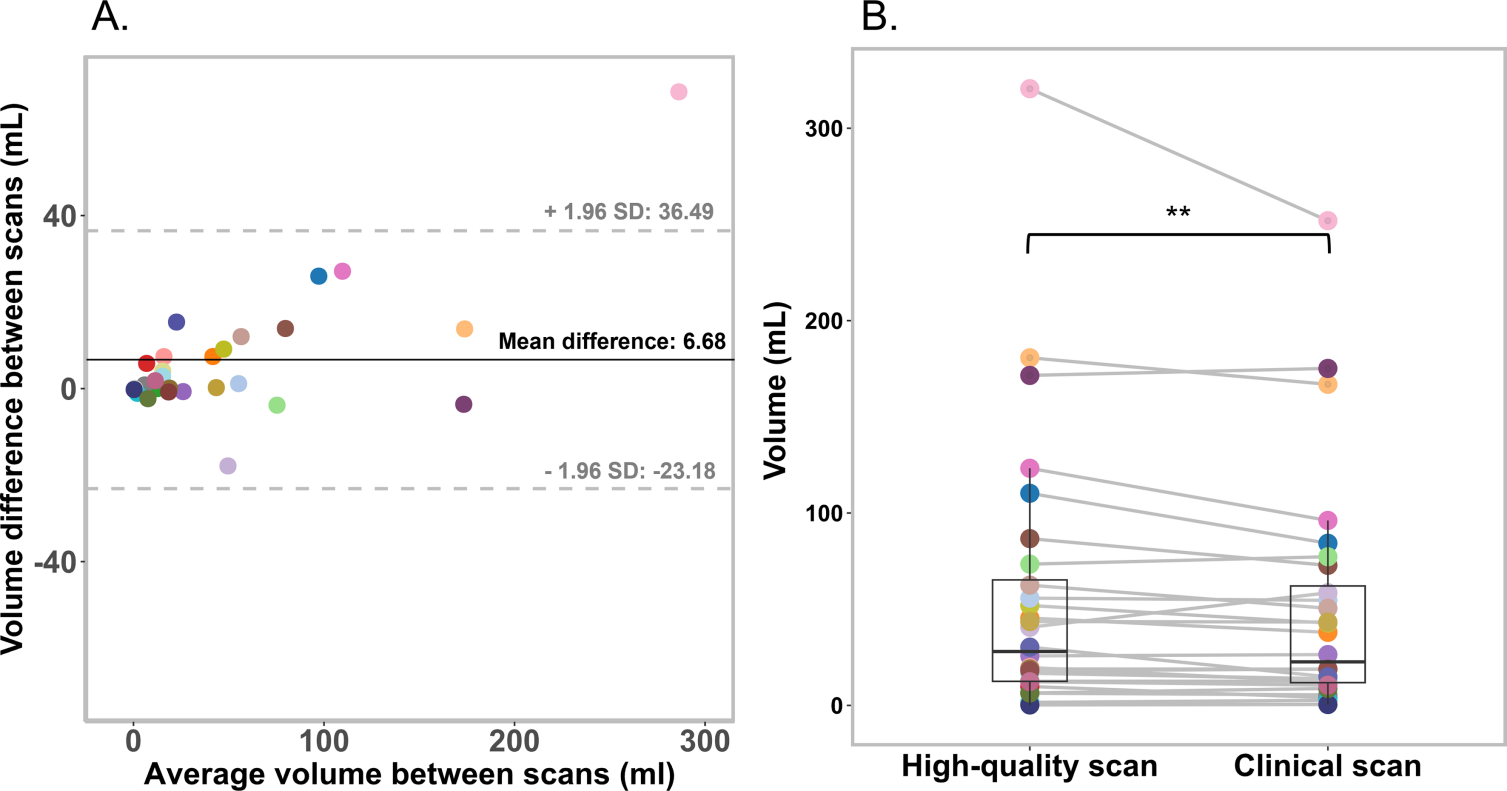
(A) Bland–Altman plot illustrating the differences in the volume measurements between the high‐quality 7 T MRI scans and clinical MRI scans. Each individual data point represents the result from an individual patient. The limits of agreement (indicated by the grey dotted lines) illustrate the range which most differences fall into (± 1.96 of the standard deviation), whereas the black central line depicts the mean difference. (B) Box plot depicting the volume measurements per patient for both the high‐quality 7 T MRI scans and clinical MRI scans. Data points per scan correspond to the values of an individual patient, and in B, the corresponding values of a patient are connected with a line.

Volume and shape markers were compared between the high‐quality 7 T MRI scans and the clinical scans in order to quantitatively compare differences in the T_2_ hyperintense area extension. A significantly higher volume of the T_2_ hyperintense area was shown for the high‐quality 7 T MRI scans (Median: 28.00 mL, IQR ‐ 25^th^ and 75^th^ percentiles: 12.51–59.13) compared with the clinical MRI scans (24.33 mL, 11.84–56.56; *p* = 0.016), as shown in Table 2. The Bland–Altman plot also showed that the mean differences was above 0 (Figure 6).

**TABLE 2:** Difference in volume and shape markers of all patients between the high‐quality 7 T MRI scans and the clinical scans.

	High‐quality 7‐T MRI scan (median (IQR—25^th^ and 75^th^ percentiles))	Clinical MRI scan (median (IQR—25^th^ and 75^th^ percentiles))	*p* value
Volume (mL)	28 (12.51–59.13)	24.33 (11.84–56.56)	0.016
Convexity	0.55 (0.48–0.61)	0.68 (0.60–0.77)	< 0.001
Solidity	0.36 (0.31–0.49)	0.43 (0.34–0.55)	< 0.001
Concavity Index	1.59 (1.53–1.64)	1.41 (1.34–1.51)	< 0.001
Fractal Dimension	2.10 (2.06–2.15)	1.90 (1.81–1.96)	< 0.001

*Note:* Volume and shape markers are expressed as medians and the respective interquartile ranges are displayed. All parameters differed significantly between the high‐quality 7 T MRI scans and clinical scans.

Abbreviation: IQR, interquartile range.

**FIGURE 7:**
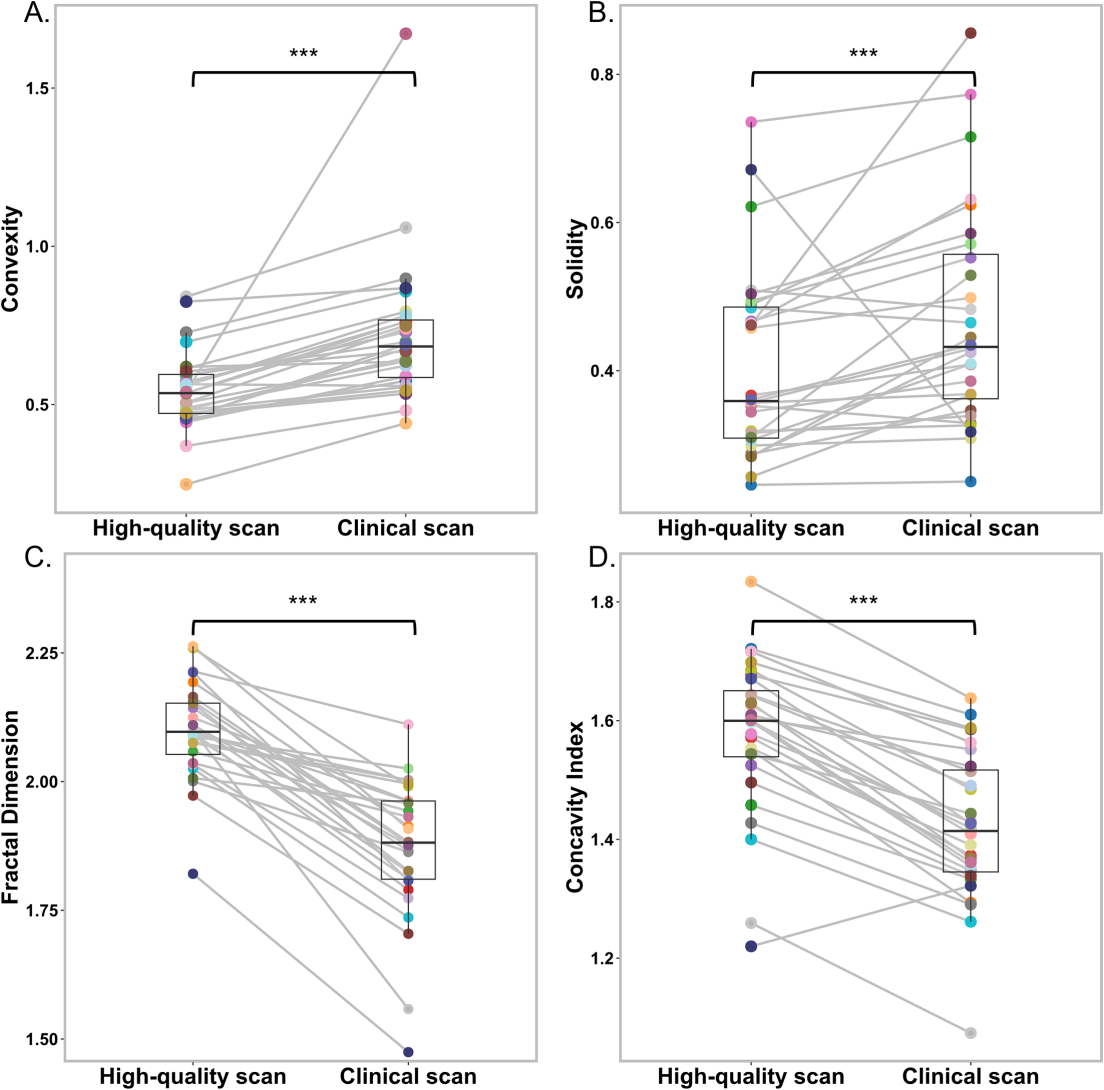
Box plots illustrating (A) convexity, (B) solidity, (C) fractal dimension, and (D) concavity index shape marker values for both the high‐quality 7 T MRI scans and the clinical scans. Data points per scan are connected between the high‐quality 7 T MRI and clinical MRI scans and correspond to the values of an individual patient. All markers differed significantly between scans (*p* > 0.001).

We also identified a statistically significant more complex shape of the T_2_ hyperintense areas on the high‐quality 7 T MRI scans compared with the clinical scans (all *p* < 0.001). These results are depicted in Figure 7, where the T_2_ hyperintense areas in the high‐quality 7 T MRI scans show a significantly lower convexity and solidity (Figure 7A,B, *p* < 0.001), as well as a higher fractal dimension and concavity index (Figure 7C,D, *p* < 0.001), all indicating a more complex shape. In Figure S3, Bland–Altman plots are shown that support these findings. In sensitivity analyses, we observed that excluding lesions < 10 cm^3^ did not impact our results, and the results obtained remained very similar to when all the patients were included in our analysis (Table S1).

## 4 Discussion

In the current study, we show that high‐quality 7 T MRI scans may provide more detail on the extension, size, and complexity of the T_2_ hyperintense areas in patients with a glioma. We showed extension of the T_2_ hyperintense areas via the corpus callosum to the opposite hemisphere in 4 patients on the high‐quality 7 T scans that was not visible on the clinical scan. Furthermore, we found a significantly larger volume of the T_2_ hyperintense areas on the high‐quality scans compared with the clinical scans. We also found a higher complexity of the T_2_ hyperintense areas on the high‐quality 7 T MRI scans compared with the clinical scans.

We wanted to exploit the higher resolution that can be obtained with T_2_WI hypothesizing that this would allow for increased visualization of details on 7 T MRI. Although this study was focused on T_2_ hyperintensities in glioma patients, other modalities such as SWI and contrast enhanced MRI have their own role in diagnosis and follow‐up of glioma, albeit beyond our current focus. When T_2_WI scans at clinical field strengths were not available, we used T_2_‐FLAIR scans instead. Despite the lower resolution of the T_2_‐FLAIR scans compared with T_2_WI (in our case due to increased slice thickness), it is commonly used in clinical practice given the advantage of free fluid suppression. We have also assessed the shape of the T_2_ hyperintense areas in glioma patients and investigated whether there is a difference in determining lesion complexity between 7 T MRI scans and conventional MRI scans. To the best of our knowledge, this study is the first to study the shape of T_2_ hyperintense areas in gliomas. This shape has, however, been previously studied in other pathologies with a similar methodology [9, 10].

Our study shows that there is an improvement in T_2_ hyperintense area visualization with 7T high‐quality MR images. More specifically tissue boundaries could be better discerned and the involvement of brain structures such as the corpus callosum and the optical tract could be more clearly identified. For instance, the clinical scan of the patient in Figure 5 did not clearly show whether the optical tract was connected to the tumor T_2_hyperintese area (due to partial volume effects), but this was clearly visible on the 7 T high‐quality scan. These findings are in line with previous studies where high‐quality 7 T MRI T_2_WI, allowed for improved visualization of disease specific changes, as well as improved visualization of different anatomical brain structures. For example, in the case of multiple sclerosis, the T_2_WI 7 T scan has been shown to allow improved visualization of gray and white matter lesions and other structural abnormalities [11]. Similarly, in patients with tuberous sclerosis complex, 7 T MRI facilitated the visualization of microtubers and radial glial abnormalities providing improved characterization of the lesion margins. The enhanced visualization of subtle margins could support pathological findings that had only been observed in animal‐models before [12]. Another interesting finding has been that different segments of the globus pallidus in the brain could be more clearly depicted. Specifically, whereas the 3 T T_2_WI could hardly show the medial medullary lamina and accessory medullary lamina, in the 7 T T2WI both structures could be clearly distinguished. [13] In gliomas, only few previous studies were performed with 7 T MRI brain scans. Our results showed that with the 7 T high‐quality T_2_WI images, T_2_ hyperintense areas extension were significantly larger and their shape was significantly more complex compared with conventional MRI scans. Our findings are surprisingly not in line with the previous study of Regnery et al., which concluded that the gross tumor volume measured at 7 T was significantly smaller than at 3 T. In their investigation, T_2_ hyperintense area measurements were compared between 3T and 7 T T_2_‐FLAIR images, which are known to be more sensitive to signal loss near the skull base and in the center of the brain (i.e., near the brainstem) [13]. The visualization and consequent measurement of tumor lesions close to those areas might become compromised using 7 T T_2_‐FLAIR. The fact that our study mostly used T_2_WI instead could explain the contradicting results. However, we observed that at least in one case (Figure S2A) the clinical T_2_WI was more robust in the lower brain regions, despite the fact that the T_2_ TSE sequence does suffer from signal loss as severely as the T_2_‐FLAIR does. In our study in some cases where a T_2_WI was not available we also resorted to T_2_‐FLAIR images. However, when leaving those cases out we did not reach different results (results not shown). We also showed that the T_2_ hyperintense areas were more complex when measured on the 7 T high‐quality scans compared with the clinical scans. This could be explained by the fact that we are able to capture more fine details of the tumor boundaries due to the smaller voxels. Enhancing the portrait of tumor boundaries may aid in understanding, for instance, its growth pattern in the brain. High‐quality imaging might possibly also aid in distinguishing nonenhancing tumor tissue from gliosis, despite both having long T_2_ relaxation times. Visual features might help in some way to further differentiate. Gliosis typically mostly shows a more homogeneous and smoother T_2_ signal compared with tumor tissue. In contrast, nonenhancing tumor parts may present with a more heterogeneous signal due to most likely variations in cellular density, as well as possible influences of neighboring edema and necrosis. Furthermore, nonenhancing tumor parts may present with mass effect especially in the cortico‐subcortical region and might involve tracts, resulting in a more irregular shape. High‐resolution T_2_‐WI helps to capture these borders more accurately and thus could help in further differentiation. On the other hand, an inherent limitation of these technique includes the inability to distinguish solid T_2_ enhancing tumor components and inflammation derived edema. The latter often results from treatment induced abnormalities. Although higher quality T_2_WI or T_2_‐FLAIR images may not entirely resolve this challenge, they do afford us a clearer understanding of tumor shape and the extent of their growth. Enhanced detailed visualization is believed to be crucial in tracking the growth of gliomas along white matter tracts, offering a more definitive indication as to whether the visualized lesion is indeed a component of solid tumor progression. Better visualization of tumor growth extension pattern could also be of added clinical value for treatment planning for radiotherapy and resection. For example, in cases where glioma T_2_ hyperintense areas are more diffuse, it could be of added value to include 7 T high‐quality T_2_WI for radiotherapy planning. Additionally, these scans could also help to determine exact tumor boundaries for surgical resection. Specifically for nonenhancing lower gliomas, where maximally safe resection is oftentimes aimed for, the improved visualization could have a significant impact on patient's prognosis, but this needs to be proven in a prospective clinical trial. Perhaps better planned radiotherapy and surgery could be less detrimental for patient's cognitive functioning and improve their quality of life. It is already known that the extent of resection significantly increases the overall survival of patients with a low‐grade glioma [14]. Lastly, the 7 T T_2_ TSE scan has a slightly longer scan duration relative to the clinical scan. However, considering its absolute total acquisition time duration of 05:12 min, it remains feasible for scanning this in a clinical setting.

The strengths of our study include a larger sample size compared with a previous study done on this topic. Additionally, our protocol included the acquisition of both T_2_WI and T_2_‐FLAIR, which could be consulted during the tumor segmentation process. On the other hand, our study also had a few limitations. The patient population that was included was heterogeneous, and consisted mostly of patients with glioblastoma and only few patients with lower grade glioma. High grade gliomas appear more diffuse, whereas lower grade glioma usually have more circumscribed lesions. Our results show that lesion boarders become better defined on the high‐quality scans, which can be of greater advantage for diffuse tumors. Lower grade tumors, because of their more circumscribed nature, might, relatively to the higher grade tumors, show less difference in defining the T_2_ hyperintense areas between the clinical and the high quality scans. However, because the number of lower grade tumor included is limited, we cannot conclude what the overall benefit would be specifically for this group of patients. Moreover, patients were included at different time points during their diagnostic and treatment workup. Although most were included at 3 months follow‐up, few were also treatment naïve or were further along in their treatment course. For example, patients who have received radiotherapy may have edema, which cannot be distinguished from solid tumor, both appearing T_2_ hyperintense, contributing to a possible overestimation of tumor size. Additionally, for a few patients there was no T_2_WI available and we had to utilize the T_2_‐FLAIR instead. Although these two imaging techniques are not exactly the same we expect that the differences between high‐quality and clinical scans remain comparable between these two techniques. Moreover, the imaging acquisition parameters from both clinical (1.5 T/3 T) and 7 T scans were not similar. The clinical scans were established through clinical consensus and were primarily utilized for patient's clinical care. Thus these thus could not be modified for our research. On the other hand we optimized the 7 T T_2_WI to achieve the most optimal images to establish especially how much of the extent of T_2_ hyperintense areas might be missed on clinical MRI scans. We acknowledge that future studies could explore optimized parameters in clinical field strengths that maximize resolution to assess how closely they can approximate 7 T images. Note that this is also the reason why we refer to the 7 T scans as ‘high‐quality’ to avoid the impression that this is a 3 T versus 7 T comparison. With the increase in field strength, there is a greater susceptibility to magnetic field inhomogeneities. Figure S2 illustrates cases where B_1_ or B_0_ inhomogeneities could have contributed to shading artifacts, primarily in the lower brain regions, which impaired visualization in these areas. Non‐uniform RF results in variations in the flip angle across the image, which can both lower signal intensities as well as affect contrast. At the same time, variations in B_0_ may cause local field inhomogeneities resulting in signal loss. Lastly, more artifacts as well as the inherent increased tissue contrast present on the high‐quality 7 T images may have impacted the quantitative geometric measurements. Notably, we consider the increase in contrast in tissue contrast an inherent part of the higher quality images, which was the basis of this investigation, that is, a wanted influence. We believe these points represents a current limitation of our 7 T imaging setup (two channel transmit head‐coil) that should be carefully considered, particularly when imaging tumors located in lower brain regions. Techniques like multitransmit can resolve most of the inhomogeneity issues.

In conclusion, our study suggests that high‐quality 7 T MRI scans may show more detail on the exact extension, size and complexity of T_2_ hyperintense areas in patients with a glioma. This information could aid in more accurate planning of treatment, such as surgery and radiotherapy, but this needs to be proven in a prospective trial.

## Supporting information


**Figure S1.** Visual assessment of T_2_ hyperintensities in the corticospinal tract using T_2_‐weighted images on A. ‐ D. the clinical MRI scans and on E. ‐ H. the high‐quality 7 T MRI scans. An example of a patient with a glioblastoma where most likely Wallerian degeneration is present due to the tumor pathology. This example illustrates how the T_2_ hyperintensities on the high‐quality images (on the bottom row) are more clearly visible, especially the lesion in D. and H., which is virtually invisible on the clinical scan, whereas its presence can be clearly visualized on the high‐quality image (D.1 and H.1, respectively). Compared to the clinical scans, the high‐quality scans show a clearer connection of the primary tumor lesion (A. & E,.) and the Wallerian degeneration along the corticospinal tract.
**Figure S2.** Four different example patients, In A. clinical and E. high‐quality T_2_ weighted scans from a glioblastoma patient who has had partial tumor resection, chemo‐ and radiotherapy. In B. – D. clinical and F. – H. high‐quality T_2_‐FLAIR scans, where each column represents one patient with a glioblastoma, anaplastic astrocytoma and a glioblastoma, respectively. Regarding treatment, these patients have had a biopsy, partial tumor resection with chemo‐ and radiotherapy and a biopsy, respectively. These examples illustrate cases where the clinical scans shows to be superior than the 7 T high‐quality ones. In the lower row we can see a drop in signal around the center of the brain. The hypointense regions make it challenging to visualize and correctly assess the involvement and extension of T_2_ hyperintense areas in those areas.
**Figure S3.** Bland–Altman plots that illustrate the difference between the shape marker measurements calculated from the high‐quality 7 T MRI scans and the clinical MRI scans. Each individual data point represents the result from one individual patient. The limits of agreement (indicated by the grey dotted lines) illustrate the range that most differences fall into (± 1.96 of the standard deviation), while the black central line depicts the mean difference. Most data points lie between the limits of agreement.
**Table S1.** Difference in volume and shape markers of patients (*n* = 22) with lesions > 10 cm^3^ between the high‐quality 7 T MRI scans and the clinical scans.

## Data Availability

The data that support the findings of this study can be made available upon reasonable request.
