# Supplementary material for: Extension of T2 Hyperintense Areas in Patients With a Glioma: A Comparison Between High‐Quality 7 T MRI and Clinical Scans
Source: NMR Biomed. 2025 Jan 28;38(3):e5316. doi: 10.1002/nbm.5316 (PMC11775408; doi:10.1002/nbm.5316)
Supplement: Supplementary file 1 — Figure S1. Visual assessment of T2 hyperintensities in the corticospinal tract using T2‐weighted images on A. ‐ D. the clinical MRI scans and on E. ‐ H. the high‐quality 7 T MRI scans. An example of a patient with a glioblastoma where most likely Wallerian degeneration is present due to the tumor pathology. This example illustrates how the T2 hyperintensities on the high‐quality images (on the bottom row) are more clearly visible, especially the lesion in D. and H., which is virtually invisible on the clinical scan, whereas its presence can be clearly visualized on the high‐quality image (D.1 and H.1, respectively). Compared to the clinical scans, the high‐quality scans show a clearer connection of the primary tumor lesion (A. & E,.) and the Wallerian degeneration along the corticospinal tract. Figure S2. Four different example patients, In A. clinical and E. high‐quality T2 weighted scans from a glioblastoma patient who has had partial tumor resection, chemo‐ and radiotherapy. In B. – D. clinical and F. – H. high‐quality T2‐FLAIR scans, where each column represents one patient with a glioblastoma, anaplastic astrocytoma and a glioblastoma, respectively. Regarding treatment, these patients have had a biopsy, partial tumor resection with chemo‐ and radiotherapy and a biopsy, respectively. These examples illustrate cases where the clinical scans shows to be superior than the 7 T high‐quality ones. In the lower row we can see a drop in signal around the center of the brain. The hypointense regions make it challenging to visualize and correctly assess the involvement and extension of T2 hyperintense areas in those areas. Figure S3. Bland–Altman plots that illustrate the difference between the shape marker measurements calculated from the high‐quality 7 T MRI scans and the clinical MRI scans. Each individual data point represents the result from one individual patient. The limits of agreement (indicated by the grey dotted lines) illustrate the range that most differences [file NBM-38-e5316-s001.docx]

**Supplementary Material**

**
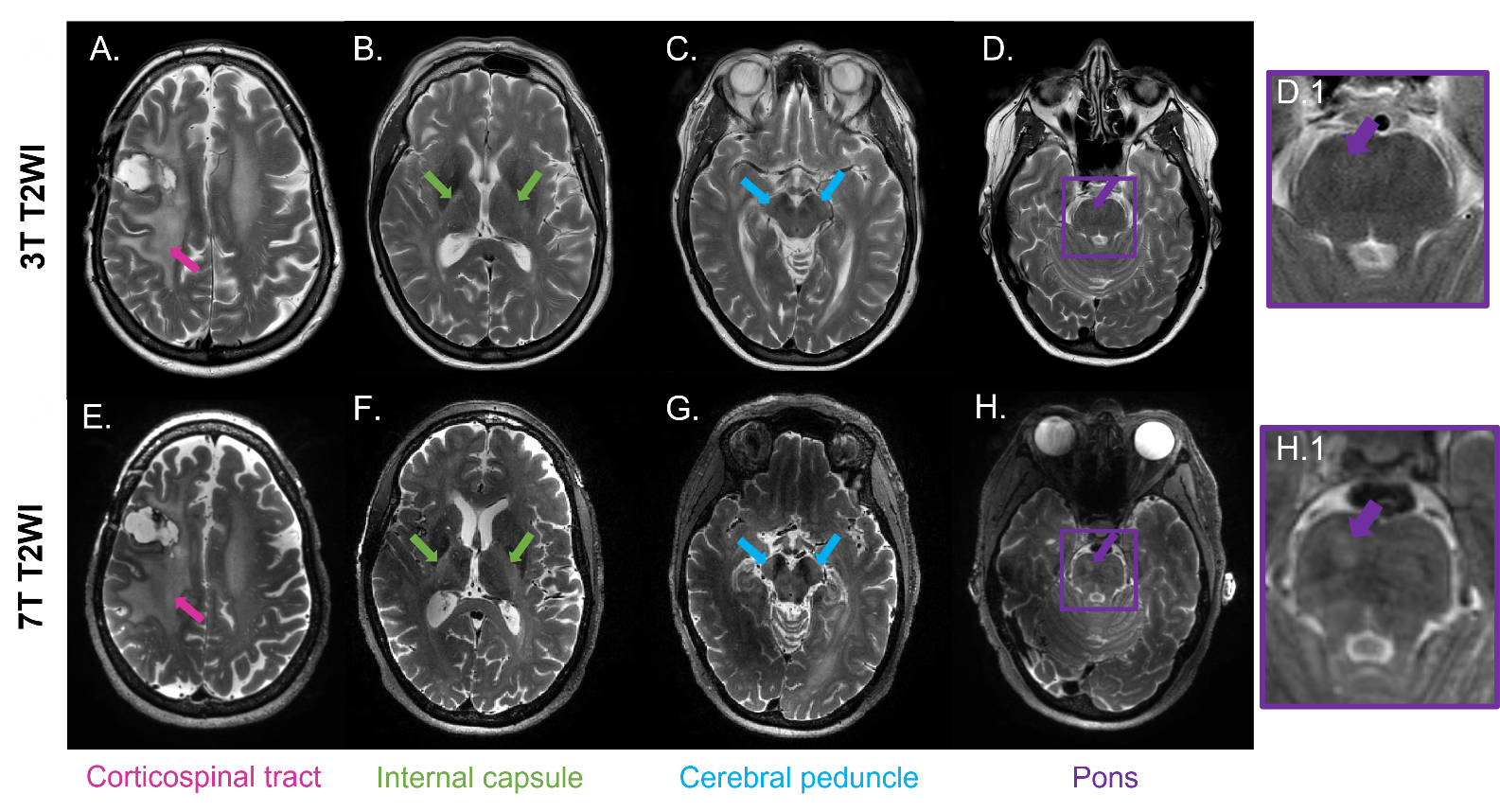
**

Supplementary Figure S1. Visual assessment of T2 hyperintensities in the corticospinal tract using T2-weighted images on A. - D. the clinical MRI scans and on E. - H. the high-quality 7 T MRI scans. An example of a patient with a glioblastoma where most likely Wallerian degeneration is present due to the tumor pathology. This example illustrates how the T2 hyperintensities on the high-quality images (on the bottom row) are more clearly visible, especially the lesion in D. and H., which is virtually invisible on the clinical scan, whereas its presence can be clearly visualized on the high-quality image (D.1 and H.1, respectively). Compared to the clinical scans, the high-quality scans show a clearer connection of the primary tumor lesion (A. & E.) and the Wallerian degeneration along the corticospinal tract.


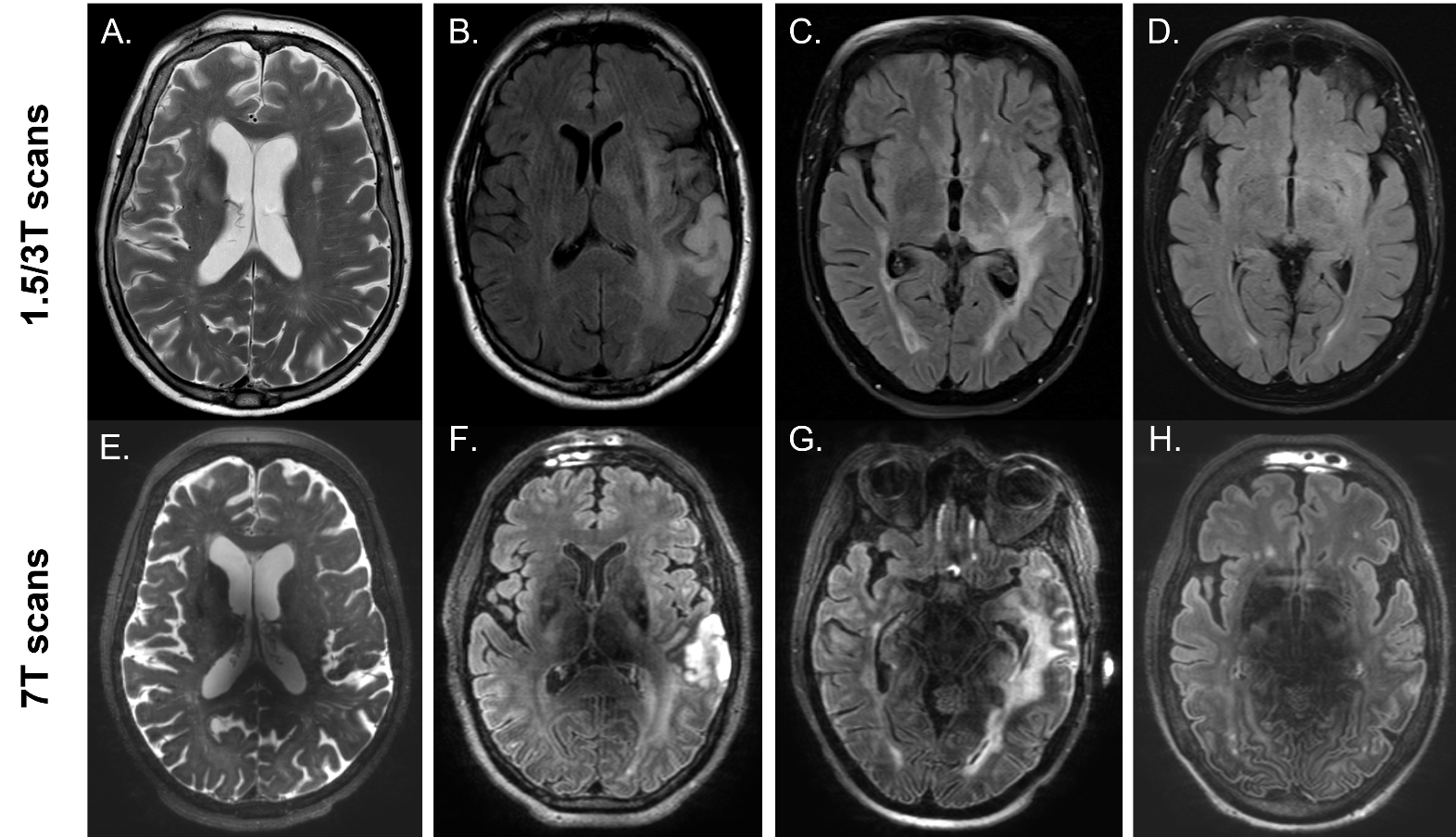


Supplementary Figure S2. Four different example patients, In A. clinical and E. high-quality T2 weighted scans from a glioblastoma patient who has had partial tumor resection, chemo- and radiotherapy. In B. – D. clinical and F. – H. high-quality T2-FLAIR scans, where each column represents one patient with a glioblastoma, anaplastic astrocytoma and a glioblastoma, respectively. Regarding treatment, these patients have had a biopsy, partial tumor resection with chemo- and radiotherapy and a biopsy, respectively. These examples illustrate cases where the clinical scans shows to be superior than the 7 T high-quality ones. In the lower row we can see a drop in signal around the center of the brain. The hypointense regions make it challenging to visualize and correctly assess the involvement and extension of T2 hyperintense areas in those areas.


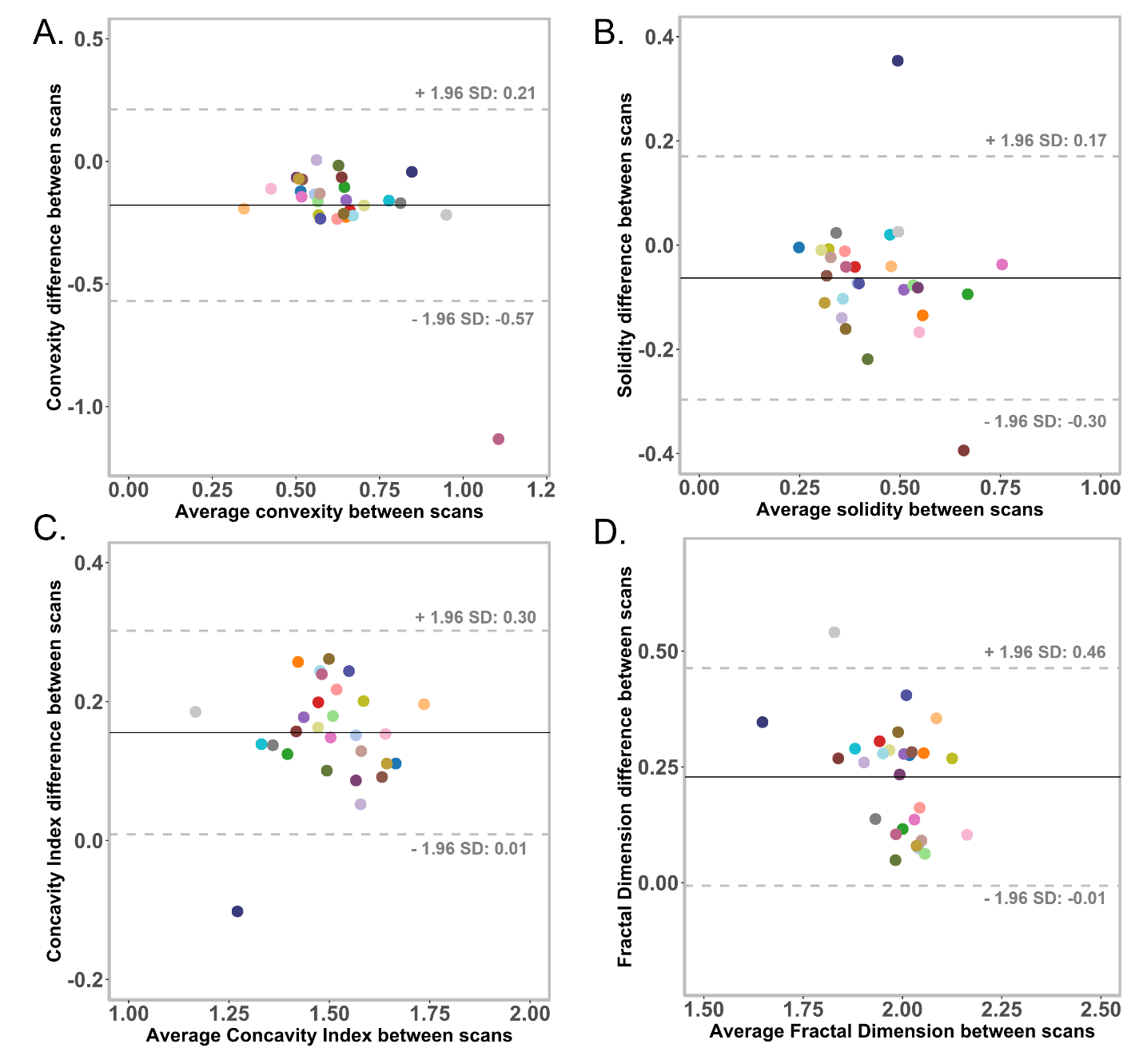


Supplementary Figure S3. Bland-Altman plots that illustrate the difference between the shape marker measurements calculated from the high-quality 7 T MRI scans and the clinical MRI scans. Each individual data point represents the result from one individual patient. The limits of agreement (indicated by the grey dotted lines) illustrate the range that most differences fall into (± 1.96 of the standard deviation), while the black central line depicts the mean difference. Most data points lie between the limits of agreement.


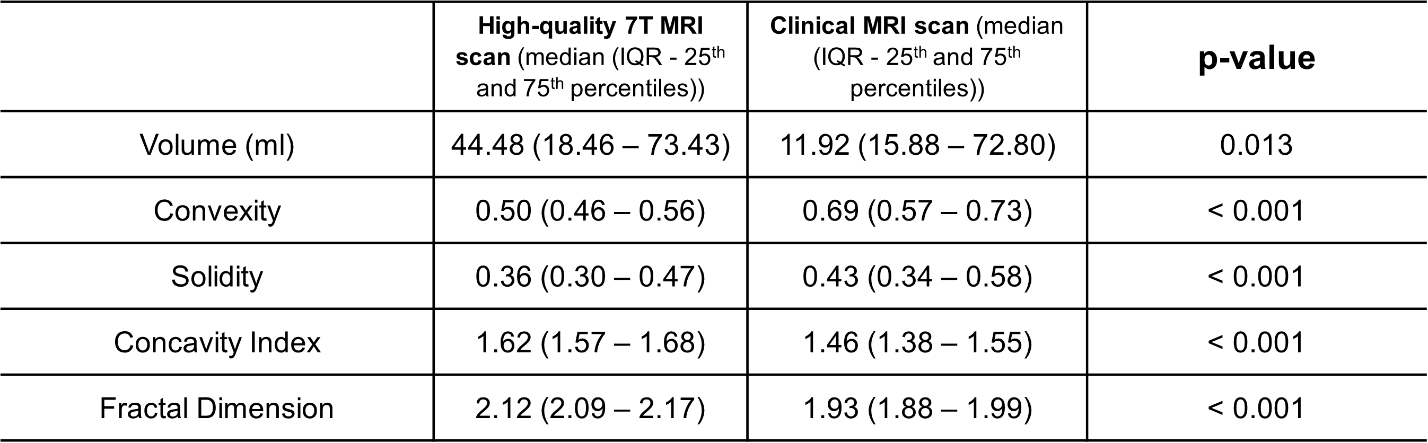

**Supplementary Table 1.** Difference in volume and shape markers of patients (n= 22) with lesions >10 cm^3^ between the high-quality 7T MRI scans and the clinical scans.

Volume and shape markers are expressed as medians and the respective interquartile ranges are displayed. All parameters differed significantly between the high-quality 7 T MRI scans and clinical scans. IQR: interquartile range
